# Case report of bilateral relapsing-remitting sciatic nerve palsy during two pregnancies

**DOI:** 10.1186/s13104-015-1647-1

**Published:** 2015-11-06

**Authors:** Sonja Suntrup-Krueger, Matthias Schilling, Wolfram Schwindt, Heinz Wiendl, Sven G. Meuth

**Affiliations:** Department of Neurology, University Hospital Muenster, Albert-Schweitzer-Campus 1, Gebäude A1, 48149 Münster, Germany; Department of Clinical Radiology, University Hospital Muenster, Albert-Schweitzer-Campus 1, Gebäude A1, 48149 Münster, Germany

**Keywords:** Sciatic nerve palsy, Pregnancy, Hormone-dependent neoplasm, Intraneural perineurioma, Immunoglobuline therapy

## Abstract

**Background:**

Unlike puerperal peripheral nerve lesions, mononeuropathy during pregnancy is rarely encountered. We report a case of bilateral relapsing-remitting sciatic nerve palsy during two pregnancies. An extensive literature search in PubMed brought no similar cases.

**Case presentation:**

A healthy young woman presented with initially unilateral sciatic nerve palsy, which manifested and worsened during the early phases of two successive pregnancies. Electrophysiology revealed axonal lesion of the sciatic nerve with predominant affection of the peroneal part. Extensive laboratory examination including cerebrospinal fluid examination was unremarkable. MR imaging was compatible with bilateral intraneural perineurioma. Recurrent occurrence during two pregnancies and an anamnestic relationship between intermediate worsening of the paresis and the menstrual cycle suggested hormone-dependency of the tumor. However, response to repeated intravenous immunoglobuline (IVIG) therapy during pregnancy and shortly after childbirth resulted in partial reversion of foot drop. This was also indicative of an immunoneuropathy. Nerve biopsy was not performed because of clinical improvement. The precise underlying neuropathological mechanism remained unclear.

**Conclusion:**

To increase knowledge and awareness of this rare entity, potential etiologies of mononeuropathies during pregnancy are discussed in the context of this case report. In the rare occasion of peripheral nerve mononeuropathy during pregnancy, in which therapeutic opportunities are limited, IVIG therapy may be an option when the etiology cannot clearly be determined after thorough medical investigation.

## Background

Whereas postpartum traumatic neuropathy is a known entity, mononeuropathy during pregnancy is rarely encountered. Here we report an unusual case of bilateral sciatic neuropathy manifesting and worsening in early pregnancy, respectively. An extensive literature search in PubMed brought no similar cases. On this occasion potential etiologies of mononeuropathies during pregnancy are discussed in the context of our case report.

## Case presentation

A 28-year-old healthy woman with unremarkable medical history except for mild hypothyreosis developed sudden painless left-sided foot drop during her first pregnancy at 16 weeks of gestation. Lumbar nerve root compression was excluded by MRI. Electroneurographic and electromyographic examination 6 weeks after symptom onset revealed an isolated axonal lesion of the sciatic nerve with predominant affection of the peroneal portion. There was no history of trauma. Family history was negative for neurofibromatosis type 1, hereditary motor and sensory neuropathy or other neuromuscular diseases. She was diagnosed as sciatic nerve palsy of unknown origin and was recommended for physiotherapy (strengthening exercises to the left tibialis anterior muscle). Paresis persisted during the further course of her pregnancy. Additional neurologic symptoms did not occur. Sensory deficits were absent. She had an uneventful Caesarean delivery at 40 gestational weeks and gave birth to a healthy girl. Thorough neurologic investigation was performed three weeks later. Follow-up MRI of the lumbar spine was again normal. Routine laboratory parameters were unremarkable. Cerebrospinal fluid examination including immunologic and infectiologic parameters was within normal limits except for a slightly increased protein level (730.4 mg/l). Inflammatory neuropathy was assumed. However, application of intravenous immunoglobulins (IVIG) for 3 days had no beneficial effect at this time. The patient got bracing for her left leg and further physiotherapy was prescribed.

Follow-up examination one year later showed ongoing weakness of the left foot (grade 2/5) and toe extension (grade 3/5) as well as ankle eversion (grade 2/5). Electroneurography demonstrated stable findings with prolonged distal motor latency of the tibialis and peroneus nerve and marked reduction of amplitudes in the peroneus nerve. A causal relation between paresis and pregnancy was considered highly unlikely at that point in time.

**Fig. 1:**
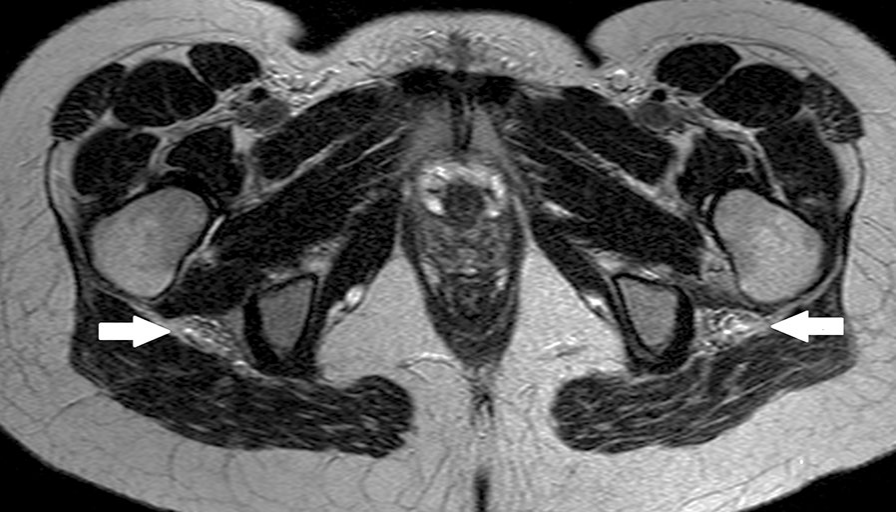
Axial T2-weighted MRI of the pelvis. It shows bilateral enlarged sciatic nerves (*arrows*) with increased signal intensity. Note that single fascicles are separated from each other but the fascicular structure of the nerve is preserved

Three years after the first presentation at the time of her second pregnancy she reported that the residual paresis of her left leg had gradually worsened again since the 8th week of gestation. Moreover, she had experienced slight weakness of foot extension in the other leg as well. Upon admission at 28 gestational weeks, neurological examination revealed complete palsy of left foot and toe extension as well as ankle eversion and slight weakness of ankle inversion and plantar flexion (grade 4/5). The right leg now also showed paresis of foot and toe extension (grade 4+/5). Sensation was normal. The patient did not report any pain. Motor nerve conduction studies confirmed deterioration with conduction now being absent in the left peroneal nerve. Detailed history taking disclosed a relationship between intermediate worsening of the paresis and the menstrual cycle (deterioration 2–3 days prior to menstruation). MRI of the pelvis (performed without contrast agent because of pregnancy) revealed bilateral fusiform irregular expansion of the sciatic nerves with separation of single nerve fascicles and hyperintensity on T2-weighted images (Fig. 1). Compression of the nerves by other anatomical structures could be excluded. Findings were considered compatible with bilateral intraneural perineurioma. To rule out other differential diagnoses numerous laboratory exams were performed including CA-125 and Human Epididymis Protein 4 (HE4) as markers for active endometriosis or ovarial tumors as well as anti-ganglioside and glutamic acid decarboxylase (GAD) autoantibodies indicating immune-mediated neuropathy. Results were unremarkable. Repeated cerebrospinal fluid examination with flow cytometry was normal. Protein level was now within normal limits.

Because therapeutic options during pregnancy were limited and an immunological etiology was still considered, intravenous steroid therapy with 1000 mg methylprednisolone was administered for three consecutive days. It had no beneficial effect. Four weeks later the patient was additionally treated with a dose of 120 g of IVIG over 4 days. After that she reported slight improvement in gait. Standing on tip-toe was again possible. Because of that, IVIG therapy was repeated at the same dose immediately after the uneventful Caesarean section, which was performed at 38 gestational weeks.

On follow-up examination four weeks postpartum, sciatic nerve palsy had improved up to the degree of the residual paresis after her first pregnancy. Right-sided foot drop had resolved completely. Another MRI scan of the pelvis, now performed with contrast agent, showed a stable extent of the bilateral lesions. Uptake of contrast agent was not seen. We suggested performing a nerve biopsy to clarify the entity of the lesion and recommended another clinical, radiological and electrophysiological follow-up examination at three months to the patient. However, she refused any further diagnostics because she was content with her current state.

## Discussion

Here we report an unusual case of bilateral sciatic nerve palsy manifesting and worsening in early pregnancy, respectively. The radiological picture was compatible with bilateral intraneural perineurioma of the peroneal portion of the sciatic nerve. The occurrence during a period of hormonal change and the anamnestic relationship between intermediate worsening of the paresis and the menstrual cycle suggested hormone-dependency of the tumor. Response to timely IVIG therapy, however, was also indicative of an immunoneuropathy. An extensive literature search in PubMed for reports of lower extremity neuropathy during pregnancy brought no similar cases except for a short report of bilateral femoral neuropathy, whose etiology could also not be determined [1].

Among puerperal traumatic neuropathies, the peroneal portion of the sciatic nerve is most frequently affected [2]. Potential etiologic mechanisms include mechanical trauma through intrapelvic compression by the fetus, stretching of the nerves or pressure-induced ischaemia during labor [3]. However, mononeuropathy during pregnancy is rarely encountered. In our case compression by the fetus seemed unlikely given the fact that symptoms manifested in early pregnancy. Compression of the sciatic nerve caused by gluteal varicosis has also been described [4], but this was excluded by MRI. Intraspinal or peripheral nerve endometriosis manifestations have been reported as a cause of recurrent hormone-dependent leg monoparesis in young women [5], but endometriosis is known to be inactive during pregnancy and respective laboratory examinations were unremarkable.

Neurogenic tumors occur predominantly in young women. Neurinoma, composed largely of Schwann cells, enlarge during pregnancy presumably as a result of hormonal change and shrink after giving birth [6, 7]. Progesterone receptor expression in neurofibromas has been reported [8]. In a mouse model, estrogen and progesterone were found to foster the growth of malignant peripheral nerve sheath tumor xenografts [9]. These observations support the hypothesis about hormonal dependence of tumors originating from the neural supporting tissues. Concentrations of steroid hormones estradiol and progesterone increase during pregnancy and plasma progesterone levels are highest in the luteal phase at the end of the menstrual cycle. This is in accordance with the patient’s observation of recurrent temporary worsening of the paresis a few days before her menstruation.

Intraneural perineurioma, which was suspected in our case because of characteristic MRI findings mentioned above [10], is known to cause painless motor mononeuropathy with sparing of sensory fibers [11, 12]. Moreover, the sciatic or common peroneal nerves are the most commonly involved sites [13]. However, bilateral occurrence is extremely rare and hormone-dependent growth of this distinct entity of perineural tissue tumors has not been described before. Unlike neurofibroma it is not a Schwann cell neoplasm but the tumor arises from the perineurial layer instead. Immunohistochemical differentiation is possible because intraneural perineuriomas are positive for EMA (epithelial membrane antigen) and negative for S-100, whereas Schwann cell lesions demonstrate the opposite pattern [11]. However, in our case nerve biopsy was not performed to avoid deteriorating partially recovered nerve function after IVIG therapy.

Further uncommon pathologies of sciatic nerve tumors and tumor-like lesions consist of neurolymphoma, commonly in the scenario of a known hematologic malignancy, or amyloidosis, which is usually also systemic with multiple nerves being affected [13].

Fibromatosis and Charcot-Marie-Tooth disease were also discussed as differential diagnoses but family history was negative for both. The patient did not report any liability to pressure palsies. We considered it as highly unlikely that a hereditary neuropathy manifests and recovers so rapidly and during pregnancy only. A hereditary neuropathy possibly also would not respond to IVIG therapy.

Chronic Inflammatory Demyelinating Polyneuropathy (CIDP) and Lewis Sumner Syndrome may be considered because the radiological picture can be similar [13] and IVIG therapy was beneficial. However, electrophysiology revealed predominantly axonal lesion of motor fibers only and CSF protein level was only slightly increased if at all. Moreover, disease progression solely in phases of hormonal change is not a typical characteristic of CIDP, neither of the aforementioned entities. In summary, the precise neuropathological mechanism underlying this unusual case of relapsing-remitting sciatic nerve palsy during early pregnancy remains unclear.

## Conclusion

In the rare occasion of peripheral nerve mononeuropathy during pregnancy, in which therapeutic opportunities are limited, IVIG therapy may be an option when the etiology cannot clearly be determined after thorough medical investigation including electrophysiological, laboratory and radiological assessment.

## Consent

Written informed consent was obtained from the patient for publication of this case report and any accompanying images.
